# Simulation of winter wheat response to variable sowing dates and densities in a high-yielding environment

**DOI:** 10.1093/jxb/erac221

**Published:** 2022-06-21

**Authors:** Sibylle Dueri, Hamish Brown, Senthold Asseng, Frank Ewert, Heidi Webber, Mike George, Rob Craigie, Jose Rafael Guarin, Diego N L Pequeno, Tommaso Stella, Mukhtar Ahmed, Phillip D Alderman, Bruno Basso, Andres G Berger, Gennady Bracho Mujica, Davide Cammarano, Yi Chen, Benjamin Dumont, Ehsan Eyshi Rezaei, Elias Fereres, Roberto Ferrise, Thomas Gaiser, Yujing Gao, Margarita Garcia-Vila, Sebastian Gayler, Zvi Hochman, Gerrit Hoogenboom, Kurt C Kersebaum, Claas Nendel, Jørgen E Olesen, Gloria Padovan, Taru Palosuo, Eckart Priesack, Johannes W M Pullens, Alfredo Rodríguez, Reimund P Rötter, Margarita Ruiz Ramos, Mikhail A Semenov, Nimai Senapati, Stefan Siebert, Amit Kumar Srivastava, Claudio Stöckle, Iwan Supit, Fulu Tao, Peter Thorburn, Enli Wang, Tobias Karl David Weber, Liujun Xiao, Chuang Zhao, Jin Zhao, Zhigan Zhao, Yan Zhu, Pierre Martre

**Affiliations:** LEPSE, Univ. Montpellier, INRAE, Institut Agro Montpellier, Montpellier, France; The New Zealand Institute for Plant & Food Research Limited, Christchurch, New Zealand; Department of Life Science Engineering, Digital Agriculture, Technical University of Munich, Freising, Germany; Institute of Crop Science and Resource Conservation INRES, University of Bonn, Bonn, Germany; Leibniz Centre for Agricultural Landscape Research, Müncheberg, Germany; Leibniz Centre for Agricultural Landscape Research, Müncheberg, Germany; Brandenburg University of Technology, Faculty of Environment and Natural Sciences, Cottbus, Germany; The New Zealand Institute for Plant & Food Research Limited, Christchurch, New Zealand; Foundation for Arable Research, Templeton, New Zealand; Agricultural & Biological Engineering Department, University of Florida, Gainesville, FL, USA; Center for Climate Systems Research, Earth Institute, Columbia University, New York, NY, USA; NASA Goddard Institute for Space Studies, New York, NY, USA; International Maize and Wheat Improvement Center (CIMMYT), Mexico DF, Mexico; Institute of Crop Science and Resource Conservation INRES, University of Bonn, Bonn, Germany; Leibniz Centre for Agricultural Landscape Research, Müncheberg, Germany; Department of Agronomy, Pir Mehr Ali Shah Arid Agriculture University, Rawalpindi, Pakistan; Department of Agricultural Research for Northern Sweden, Swedish University of Agricultural Sciences Umeå, Sweden; Department of Plant and Soil Sciences, Oklahoma State University, Stillwater, OK, USA; Department of Earth and Environmental Sciences, Michigan State University, East Lansing, MI, USA; W. K. Kellogg Biological Station, Michigan State University, East Lansing, MI, USA; National Institute of Agricultural Research (INIA), Colonia, Uruguay; Tropical Plant Production and Agricultural Systems Modelling (TROPAGS), University of Göttingen, Göttingen, Germany; Department of Agroecology, Aarhus University, Tjele, Denmark; Institute of Geographical Sciences and Natural Resources Research, Chinese Academy of Science, Beijing, China; Plant Sciences Axis – Crop Science, Gembloux Agro-Bio Tech, University of Liege, Gembloux, Belgium; Leibniz Centre for Agricultural Landscape Research, Müncheberg, Germany; IAS-CSIC & DAUCO, University of Cordoba, Cordoba, Spain; Department of Agriculture, food, environment and forestry (DAGRI), University of Florence, Florence, Italy; Institute of Crop Science and Resource Conservation INRES, University of Bonn, Bonn, Germany; Agricultural & Biological Engineering Department, University of Florida, Gainesville, FL, USA; IAS-CSIC & DAUCO, University of Cordoba, Cordoba, Spain; Institute of Soil Science and Land Evaluation, University of Hohenheim, Stuttgart, Germany; CSIRO Agriculture and Food, Brisbane, Queensland, Australia; Agricultural & Biological Engineering Department, University of Florida, Gainesville, FL, USA; Institute for Sustainable Food Systems, University of Florida, Gainesville, FL, USA; Leibniz Centre for Agricultural Landscape Research, Müncheberg, Germany; Tropical Plant Production and Agricultural Systems Modelling (TROPAGS), University of Göttingen, Göttingen, Germany; Global Change Research Institute, Academy of Sciences of the Czech Republic, Brno, Czech Republic; Leibniz Centre for Agricultural Landscape Research, Müncheberg, Germany; Global Change Research Institute, Academy of Sciences of the Czech Republic, Brno, Czech Republic; Institute of Biochemistry and Biology, University of Potsdam, Potsdam, Germany; Department of Agroecology, Aarhus University, Tjele, Denmark; Global Change Research Institute, Academy of Sciences of the Czech Republic, Brno, Czech Republic; Department of Agriculture, food, environment and forestry (DAGRI), University of Florence, Florence, Italy; Natural Resources Institute Finland (Luke), Helsinki, Finland; Institute of Biochemical Plant Pathology, Helmholtz Zentrum München—German Research Center for Environmental Health, Neuherberg, Germany; Department of Agroecology, Aarhus University, Tjele, Denmark; CEIGRAM, Technical University of Madrid, Madrid, Spain; Department of Economic Analysis and Finances, University of Castilla-La Mancha, Toledo, Spain; Tropical Plant Production and Agricultural Systems Modelling (TROPAGS), University of Göttingen, Göttingen, Germany; Centre of Biodiversity and Sustainable Land Use (CBL), University of Göttingen, Göttingen, Germany; CEIGRAM, Technical University of Madrid, Madrid, Spain; Rothamsted Research, Harpenden, UK; Rothamsted Research, Harpenden, UK; Centre of Biodiversity and Sustainable Land Use (CBL), University of Göttingen, Göttingen, Germany; Department of Crop Sciences, University of Göttingen, Göttingen, Germany; Institute of Crop Science and Resource Conservation INRES, University of Bonn, Bonn, Germany; Biological Systems Engineering, Washington State University, Pullman, WA, USA; Water Systems & Global Change Group, Wageningen University, Wageningen, The Netherlands; Institute of Geographical Sciences and Natural Resources Research, Chinese Academy of Science, Beijing, China; Natural Resources Institute Finland (Luke), Helsinki, Finland; CSIRO Agriculture and Food, Brisbane, Queensland, Australia; CSIRO Agriculture and Food, Canberra, Australian Capital Territory, Australia; Institute of Soil Science and Land Evaluation, University of Hohenheim, Stuttgart, Germany; College of Environmental and Resource Sciences, Zhejiang University, Hangzhou, Zhejiang, China; National Engineering and Technology Center for Information Agriculture, Key Laboratory for Crop System Analysis and Decision Making, Ministry of Agriculture, Jiangsu Key Laboratory for Information Agriculture, Jiangsu Collaborative Innovation Center for Modern Crop Production, Nanjing Agricultural University, Nanjing, China; College of Resources and Environmental Sciences, China Agricultural University, Beijing, China; Department of Agroecology, Aarhus University, Tjele, Denmark; College of Resources and Environmental Sciences, China Agricultural University, Beijing, China; CSIRO Agriculture and Food, Canberra, Australian Capital Territory, Australia; National Engineering and Technology Center for Information Agriculture, Key Laboratory for Crop System Analysis and Decision Making, Ministry of Agriculture, Jiangsu Key Laboratory for Information Agriculture, Jiangsu Collaborative Innovation Center for Modern Crop Production, Nanjing Agricultural University, Nanjing, China; LEPSE, Univ. Montpellier, INRAE, Institut Agro Montpellier, Montpellier, France; CSIRO Agriculture and Food, Australia

**Keywords:** Multi-model ensemble, sowing date, sowing density, tillering, tiller mortality, wheat, yield potential

## Abstract

Crop multi-model ensembles (MME) have proven to be effective in increasing the accuracy of simulations in modelling experiments. However, the ability of MME to capture crop responses to changes in sowing dates and densities has not yet been investigated. These management interventions are some of the main levers for adapting cropping systems to climate change. Here, we explore the performance of a MME of 29 wheat crop models to predict the effect of changing sowing dates and rates on yield and yield components, on two sites located in a high-yielding environment in New Zealand. The experiment was conducted for 6 years and provided 50 combinations of sowing date, sowing density and growing season. We show that the MME simulates seasonal growth of wheat well under standard sowing conditions, but fails under early sowing and high sowing rates. The comparison between observed and simulated in-season fraction of intercepted photosynthetically active radiation (FIPAR) for early sown wheat shows that the MME does not capture the decrease of crop above ground biomass during winter months due to senescence. Models need to better account for tiller competition for light, nutrients, and water during vegetative growth, and early tiller senescence and tiller mortality, which are exacerbated by early sowing, high sowing densities, and warmer winter temperatures.

## Teaser

An ensemble of 29 wheat crop models simulates seasonal wheat growth well under locally recommended sowing conditions, but needs improvements to capture the yield response to early sowing, especially under high sowing density.

## Introduction

Wheat is the most traded crop commodity at the global scale and provides more than 20% of calories and protein in human diets (Shiferaw *et al.*, 2013; Curtis and Halford, 2014). For decades, the demand for cereals has been increasing, driven by world population growth and dietary change (Alexandratos and Bruinsma, 2012; van Dijk *et al.*, 2021). To meet the demand, cropping systems have to increase production while facing several challenges: risks associated with climate change, limits to cropland expansion, and increasing freshwater scarcity (Eitelberg *et al.*, 2015; FAO, 2017; Gerten *et al.*, 2020). Global climate change already impacts crop growth and production through increased temperatures, changing precipitation patterns, higher extent and severity of droughts, and greater frequency of extreme events, among others (Mbow *et al.*, 2019). Genetic improvement and agronomic management adaptation, such as optimization of sowing dates and sowing rates, are promising solutions to increase the productivity of cropping systems under global climate change (Spiertz, 2012; Bai and Tao, 2017; Parent *et al.*, 2018).

Adapting sowing conditions to climate change requires an understanding of how crop growth and yield development are affected by sowing date and density. Increased temperature shortens the growing season of wheat and shifts optimal sowing dates (Tao *et al*, 2014). Shifting sowing dates and changing sowing rates affect tiller development and tiller competition (Spink *et al.*, 2000). Tillering plays an important role in crop growth and yield formation. On the one hand tillers contribute to light interception and photosynthesis, on the other hand they are reservoirs of nutrients and carbohydrates, which can be remobilized to the main stem under nutrient stress (Anderson and Garlinge, 2000). The ability to remobilize nutrients is critical during stem elongation, when the crop is growing rapidly and nutrient demand is high (Anderson and Garlinge, 2000). Therefore, tillers contribute significantly to the maintenance of ear growth and grain yield.

Process-based crop growth models were developed to study the multiple interacting factors affecting crop growth, development, and yield formation under field conditions (Chenu *et al.*, 2017). By representing the interactions between genotype, environment, and management, crop models are useful tools to identify options to improve resource use efficiency in cropping systems and narrow yield gaps (Xin and Tao, 2019; Padovan *et al.*, 2020). Crop models are also used to evaluate the impact of climate change on wheat production (Ye *et al.*, 2020) and test genetic and management adaptation pathways to maintain or increase productivity (Challinor *et al.,* 2007, 2014; Gouache *et al.*, 2012; Ruiz-Ramos *et al.* 2018). Several modelling studies have pointed out that adaptation of crop management by optimizing sowing date and density is important for maintaining high grain yields (Tao *et al.*, 2016; Parent *et al.*, 2018; Xin and Tao, 2019; Yao *et al.*, 2021).

Crop models are simplified representations of plant growth and each model is developed, tested and used under a certain range of conditions. As one moves away from the usual conditions, other mechanisms may come into play that the model does not represent. Simulating wheat growth in a high-yielding environment is already a challenge for crop models and tests their ability to achieve yields above the usual range. Changing sowing dates and densities under these conditions is an even greater challenge, and shows whether the models include the necessary mechanisms to represent these atypical environments and conditions.

Multi-model ensembles (MME) are used to simulate the effect of climate variability and change on cropping systems (Palosuo *et al.*, 2011; Asseng *et al.*, 2013; Bassu *et al.*, 2014; Webber *et al.*, 2018; Rodríguez *et al.*, 2019) and have been shown to be effective in increasing the accuracy of prediction (Martre *et al.*, 2015). Theoretical and empirical results have shown that in a wide variety of cases the ensemble mean or median is a better predictor of yield, biomass, protein, and maximum leaf area index (LAI) than individual models (Wallach *et al.*, 2018). However, the ability of MMEs to ­simulate the effect of changing sowing dates and sowing rates on yield and yield components has not yet been assessed. The objective of this study is to evaluate the accuracy of a MME of 29 process-based wheat crop models to simulate the effect of variable sowing dates and densities on wheat growth and yield in a high-yielding environment.

## Materials and methods

### Experimental dataset

The field trials were carried out by the New Zealand Institute for Plant and Food Research and the Foundation for Arable Research at two farms located at Leeston (43° 45ʹ S, 172° 15ʹ E) and Wakanui (43° 58ʹ S, 171° 48ʹ E) in the Canterbury Region of the South Island of New Zealand (Craigie *et al.*, 2015). The farm located at Wakanui has achieved the Guinness World Record for the highest wheat yield twice, in 2017 and in 2020. The local winter wheat cultivar ‘Wakanui’ was grown under non-stress conditions for six consecutive years, first at Leeston (from 2012–2013 to 2014–2015) and then at Wakanui (from 2015–2016 to 2017–2018) (see Table 1). ‘Wakanui’ is a soft wheat with very high yield potential associated with a long grain-filling period.

**Table 1.** Sowing dates, sowing densities, total irrigation and N fertilization, and summary of average weather conditions from March to December for the field experiments used in this study

Location	Growing season	Sowing dates				Sowing densities (seeds m^−2^)				Total irrigation (mm)	Total N fertilization (kg N ha^−1^)	Average daily minimum temperature (°C)	Average daily maximum temperature (°C)	Cumulative rainfall(mm)	Cumulative solar radiation (MJ m^−2^)
		Early		Locally recommended											
		February	Early March	Late March	April										
**Leeston**	2012–2013	2012-02-21		2012-03-26		50	100	150	200		138	5.4	15.6	497	3956
	2013–2014	2013-02-20^a^		2013-03-26	2013-04-16	50	100	150	200		122	6	16.1	686	3529
	2014–2015	2014-02-20	2014-03-10	2014-03-26	2014-04-23	50	100	150	200	30	188	5.9	15.9	473	3686
**Wakanui**	2015–2016	2015-02-20	2015-03-10	2015-03-20	2015-04-09			150		210	284	3.1	15.9	456	3691
	2016–2017	2016-02-24^a^	2016-03-08^a^	2016-03-29	2016-04-14			150		50	258	4.2	16.1	440	4060
	2017–2018		2017-03-09	2017-03-30	2017-04-19			150		125	270	4.1	16	811	3920

Sowing date not considered in this study because of significant lodging and diseases.

In the Canterbury region of New Zealand, winter wheat is usually sown between early April and mid-May, with some farmers sowing in late March in recent years (Craigie *et al.*, 2015). The objective of the trials was to test if sowing earlier (February or early March), and therefore increasing the canopy duration and the radiation interception, would increase grain yield. Four sowing dates were tested: February, early March, late March, and April (Table 1). At Leeston, the effect of sowing dates was studied in combination with four sowing densities: 50, 100, 150, and 200 seeds m^−2^, while at Wakanui, only the locally recommended sowing density was used (150 seeds m^−2^).

The objective of the Leeston trials was to increase wheat production in a high yielding environment without water and nitrogen stress. The trials investigated the complex interaction between tiller development, ear population, and grain yield, with the goal of finding the optimal sowing density for an early sowing date. The experiments consisted of a split-plot design with sowing dates as the main plots and sowing rates as the subplots, with four replicates. The Wakanui trials used a single sowing density and investigated different cultivars, sowing dates, and the use of plant growth regulators (2015–2016) or defoliation (2016–2017 and 2017–2018), at different sowing dates. The experiments were designed as randomized blocks with sowing dates as the main plots and cultivar by plant growth regulation or defoliation as the subplots, replicated four times. In our study, we considered only the data of the ‘Wakanui’ cultivar grown under standard growth regulation and without defoliation.

The field management was adapted each year to obtain ideal growth conditions: crops received a yearly N fertilization of between 122 and 284 kg N ha^−1^ and irrigation up to 210 mm of water.

Individual plots (12 m×1.65 m) were drilled into a top worked seed bed. At both sites, the soil type was Temuka clay loam (Fluventic Endoaquents in the USDA classification), a deep, low permeability soil with high plant available water capacity (Kear *et al.*, 1967; Craigie *et al.*, 2015). The Leeston site was characterized by a shallow water table at about 1 m below the soil surface.

Weather data were collected at a weather station located within 2 km from the experimental field and provided daily minimum and maximum temperature, rainfall, solar radiation, and wind speed and relative humidity at 2 m.

During all experiments the grain, stem, chaff, and leaf dry weight at maturity, ear number and grain number, grain unit dry weight, and harvest index were determined. The total above ground dry biomass, leaf number per stem and LAI were measured at Zadoks growth stage (Zadoks *et al*., 1974) 32 (stem elongation) and 65 (anthesis), except for the first two growing seasons of the trial. For all but the first growing season, several in-season measurements of the normalized difference vegetation index (NDVI; from Trimble Greenseeker (Trimble Agriculture Division, CO, USA) measurements) and the fraction of intercepted photosynthetically active radiation (FIPAR; from Sunscan (Delta-T devices, Cambridge, UK) measurements), as well as the date of the 32, 65, and 90 Zadoks stages were available. FIPAR measurements were particularly useful for tracking in-season changes in canopy light interception and for supplementing information on biomass and LAI that were measured only at stem elongation and anthesis. The fertile stem biomass was obtained by dividing the final total above ground biomass by the ear number, as a measure of the above ground crop biomass per ear.

### Crop growth models

Twenty-nine currently used process-based wheat crop models of the Agricultural Model Intercomparison and Improvement Project (AgMIP) Wheat group (https://agmip.org/wheat/) participated in this study and contributed to the MME output (Supplementary Table S1). Modelling groups were provided with daily weather data (minimum and maximum temperature, rainfall, solar radiation, wind speed at 2 m, relative air humidity at 2 m and average vapour pressure) and soil physico-chemical characteristics (soil water lower limit, drained upper limit, saturation, apparent bulk density, organic C and organic N concentration, and soil pH). Initial soil inorganic N amount was estimated for the upper 150 cm for each growing season, based on mineral nitrogen values measured in 2013 and 2014 in the upper 60 cm and 75 cm of soil, respectively. The soil was represented by three layers of equal thickness (50 cm) and the distribution of the total initial amount of inorganic N in each layer was estimated at 55%, 30%, and 15%, from the top layer to the bottom layer. Initial soil water content was estimated at field capacity. The same initial values of soil inorganic nitrogen and soil water content were used to initialize the simulations, regardless of sowing dates.

The models were calibrated with data measured during the 2014–2015 growing season, including a combination of four sowing dates and four sowing densities, for a total 16 different treatments. Supplied data were the mean of the four replicates. For each experiment, modellers were provided with phenological records: the date of beginning of stem extension (Zadoks 31) anthesis (Zadoks 65) and physiological maturity (Zadoks 87). In addition, the grain, stem, chaff, and leaf dry weight at maturity, ear number and grain number, grain unit dry weight, and harvest index were provided. Also, time series of measurements of total above ground dry biomass, leaf number per stem, and LAI were provided, as well as NDVI and FIPAR.

After calibration, simulations were conducted by each model for all combinations of sowing date, sowing density, and growing season (Table 1), for a total of 50 simulations. All 29 models reported above ground biomass at anthesis and maturity, grain yield, and harvest index, while LAI was reported by 28 models, grain dry weight and grain number by 15 models, and FIPAR by 13 models. The results are plotted as median and 25% and 75% quantiles of the measured and simulated variables. These values were estimated using bootstrap re-sampling.

### Crop growth model performance evaluation metrics

The interannual variability of grain yield (GY), was quantified with the coefficient of variation (CV), which is the ratio of the standard deviation (σ_GY_) to the mean ($\bar{GY}$), as a percentage. The standard deviation is given by the square root of the sum of the squared difference between the grain yield and its mean, divided by the number of grain yield values *n*.


$$\begin{array}{l} CV_{GY}=\;\frac{\sigma_{GY}}{\bar{GY}}\;\times 100 \end{array}$$ (1)

$$\begin{array}{l} \sigma_{GY}=\sqrt{\frac{\overset{n}{\underset{i=1}{\sum }}{\left(GY-\bar{GY}\right)}^{2}}{n}} \end{array}$$ (2)

The higher the coefficient of variation, the greater the level of dispersion around the mean.

The root mean square error (RMSE) is the standard deviation of the residuals and is calculated as the squared root of the mean squared difference between the observed (*O*) and the simulated (*S*) values and is given as:


$$\begin{array}{l} RMSE=\;\sqrt{\frac{\overset{n}{\underset{i=1}{\sum }}{\left(S_{i}-O_{i}\right)}^{2}}{n}} \end{array}$$ (3)

The model accuracy was quantified using the relative RMSE (RRMSE), expressed as a percentage, which is useful to compare outputs with different units. RRMSE is obtained by dividing RMSE by the mean of the observations ($\bar{O}$):


$$\begin{array}{l} RRMSE=\frac{RMSE}{\bar{O}}\times 100 \end{array}$$ (4)

We consider that model accuracy is excellent if RRMSE<10%, good if 10%<RRMSE<20%, fair if 20%≤RRMSE<30%, and poor if RRMSE≥30% (Jamieson *et al.*, 1991).

### Statistical analyses

An analysis of variance (ANOVA) was performed to test if year, sowing date, and sowing rate had significant main or interaction effects on grain yield. We considered only the Leeston trials, where both sowing date and rate were tested (Table 1). The analysis was conducted for both simulations and measurements. The ANOVA of measured grain yield was performed using data from all four replicates as independent observations (32 measurements×4 replicates=128 independent observations). The ANOVA of the simulations was conducted considering that the grain yield output of each model can be considered as an independent variable (32 simulations×29 models=928 independent observations).

## Results

### Evaluation of models and MME performance

The interactions between year, sowing date, or sowing density for grain yield were not statistically significant, either for measured or for simulated values (all *P*>0.05) but the ANOVA showed a significant variation of measured grain yield with year (*P*<0.001) and sowing date (*P*<0.01). The sowing density had no significant effect on measured grain yield (*P*=0.79; Table 2). The ANOVA of simulated grain yield showed that three main factors had a significant effect: year, sowing date, and sowing density. For both measured and simulated values year had the largest effect on grain yield, followed by sowing date, while sowing density had the lowest effect.

**Table 2.** Results from the analysis of variance for measured and simulated grain yield

Factor	Measured grain yield			Simulated grain yield		
	Degree of freedom	*F*	*P*	Degree of freedom	*F*	*P*
Year	2	15.31	<0.001	5	28.92	<0.001
Sowing date	3	5.72	<0.01	3	10.80	<0.001
Sowing density	3	0.35		3	8.68	<0.001
Year: sowing date	2	2.03		8	0.87	
Year: sowing density	6	0.16		6	0.36	
Sowing date: sowing density	9	1.90		9	0.36	
Year: sowing date: sowing density	6	0.34		6	0.04	
Residuals	96			892		

**Fig. 1.**
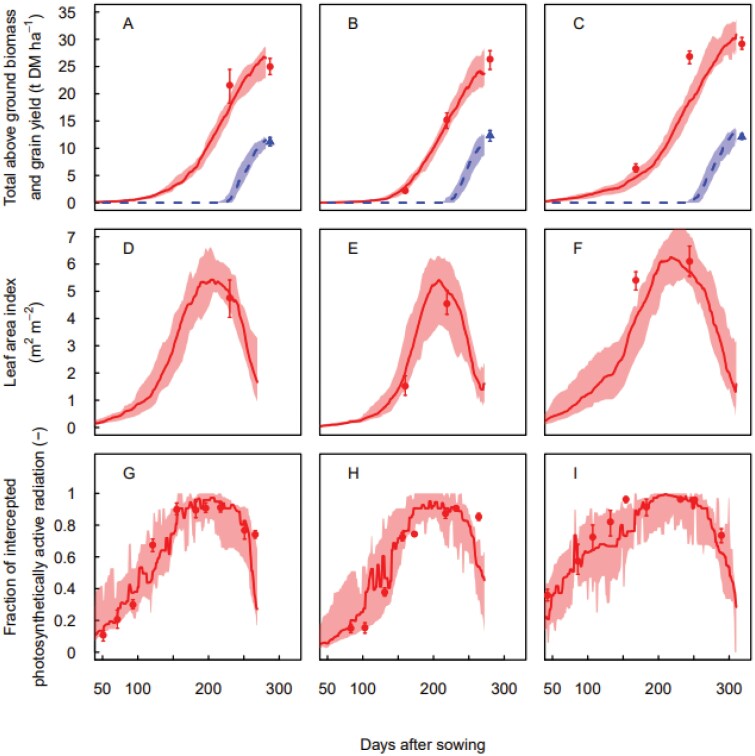
Measurements (symbols) and multi-model ensemble simulations (lines) of total above ground biomass (red circles and solid lines) and grain yield (blue triangles and dashed lines; A–C), leaf area index (D–F), and fraction of intercepted photosynthetically active radiation (G–I) versus days after sowing for the winter wheat cultivar ‘Wakanui’ sown during the locally recommended sowing window (16 April 2013 (A, D, G), 23 April 2014 (B, E, H), and 29 March 2016 (C, F, I)) and at the recommended plant density (150 plants m^−2^) in Leeston (2014 and 2015) or Wakanui (2016), New Zealand. Measured data are medians for *n*=4 independent replicates and simulated data are medians for the multi-model ensemble. Errors bars (measurements) and colour bandings (simulations) show 25%–75% quantiles.

Under locally recommended sowing conditions (sowing date between late March and April and sowing density of 150 seeds m^−2^), the MME performance was good for simulating the in-season development of the total above ground biomass, LAI and FIPAR, as well as the final grain yield measured in the field (Fig. 1). However, for winter wheat sown at late March 2016, the MME underestimated the anthesis biomass, LAI, and FIPAR at the beginning of stem elongation (Zadoks 32; Fig. 1C, F, I).

**Fig. 2.**
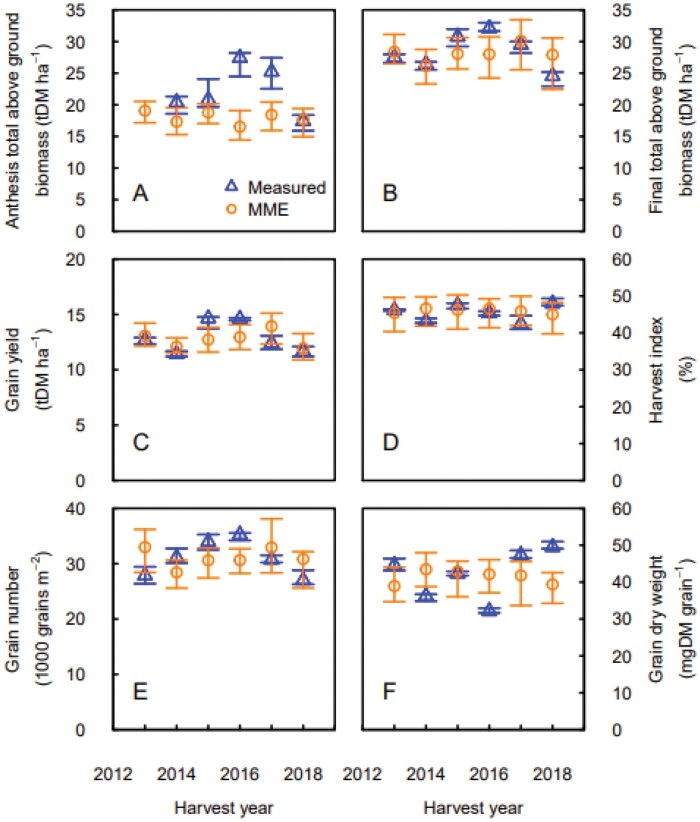
Measured (blue triangles) and simulated (orange circles) total above ground biomass at anthesis (A) and maturity (B), grain yield (C), harvest index (D), grain number (E), and grain dry mass (F) for the winter wheat cultivar ‘Wakanui’ sown in the locally recommended sowing window (late March to early April) and plant density (150 plants m^−2^) for six consecutive years in Leeston (2012–2013 to 2014–2015) then Wakanui (2015–2016 to 2017–2018), New Zealand. Measured data are medians for *n*=4 independent replicates and simulated data are medians for the multi-model ensemble, respectively. Error bars show 25%–75% quantiles.

Under locally recommended sowing conditions, the MME simulated the yearly averaged values of grain yield, final above ground biomass, and harvest index with accuracy ranging from good (RRMSE of 10.33 and 10.09 for grain yield and final biomass, respectively) to excellent (RRMSE of 5.75 for harvest index) (Supplementary Table S2; Supplementary Protocol S1; Fig. 2). Interestingly, the MME underestimated the grain yield in the calibration year (2015) while yield in early sowings was overestimated. Large discrepancies between MME simulations and measurements were found for total above ground biomass at anthesis, especially for the 2015–2016 and 2016–2017 growing seasons (Fig. 2A). These years were characterized by an underestimation of the anthesis biomass by the MME. The interannual variability of average grain dry weight was also poorly simulated by the MME (Fig. 2F).

**Fig. 3.**
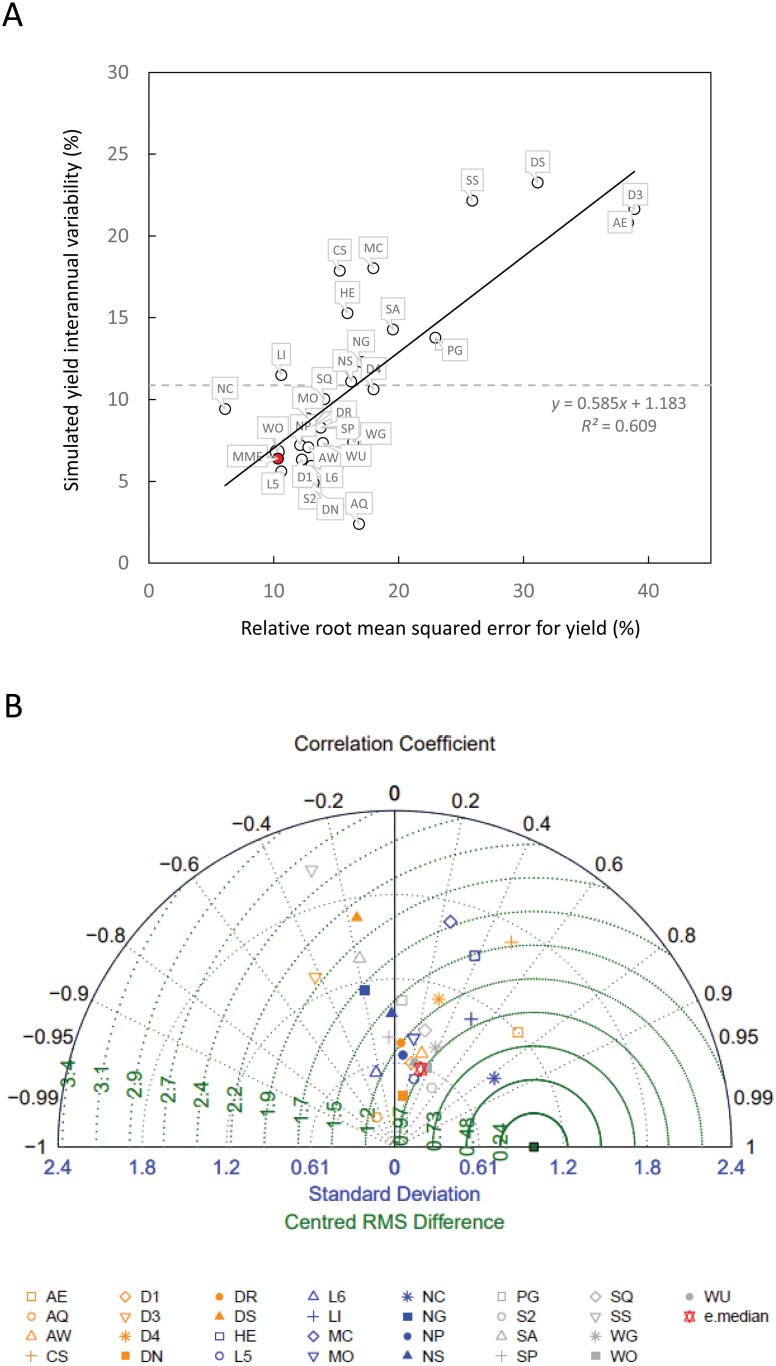
Evaluation of the performance of 29 wheat crop growth models and their ensemble for simulating grain yield. (A) Simulated grain yield interannual variability versus relative root mean square error (RRMSE) for grain yield for 29 wheat crop growth models. The horizontal dashed line indicates the measured grain yield interannual variability. (B) Taylor diagram providing the standard deviation (concentric blue lines around (0,0)), correlation (angular coordinates) and centred root mean squared error (concentric green lines) of measured grain yield (green filled square) and the 29 wheat crop growth models. In (A, B) data are for the winter wheat cultivar ‘Wakanui’ sown at the locally recommended sowing date and plant density for six consecutive years in Leeston then Wakanui, New-Zealand. Models are identified with two-letter codes (see Supplementary Table S1).

The simulated interannual variability of grain yield increased with increasing RRMSE (Fig. 3A), meaning that the models with highest RRMSE also overestimated interannual variability of yields. Interestingly one of the models with the highest RRMSE (AE) was one of the best performing models on the Taylor diagram (Fig. 3B), which does not measure bias, since the RMSE is centred by subtracting the respective means. These results show that the models match well the pattern of measured grain yields although they show a systematic shift (bias). Modelling efficiency (EF) decreased with increasing RRMSE and most models had a negative EF (Supplementary Table S2). Two models had lower mean error for grain yield than the MME (Supplementary Table S3; Fig. 3). The model with the lowest RRMSE for grain yield (NC) simulated well the interannual variability of crop growth and grain yield, while the MME underestimated it. The performance based on RRMSE and represented by the models’ rank, changed significantly when we consider only simulations of low sowing density, early sowing date or locally recommended sowing date trials (Supplementary Tables S3, S4). This highlighted that model performance varied with sowing conditions. MME simulations showed the best performance (lowest value of RRMSE) under low sowing density conditions.

### Effect of sowing density

**Fig. 4.**
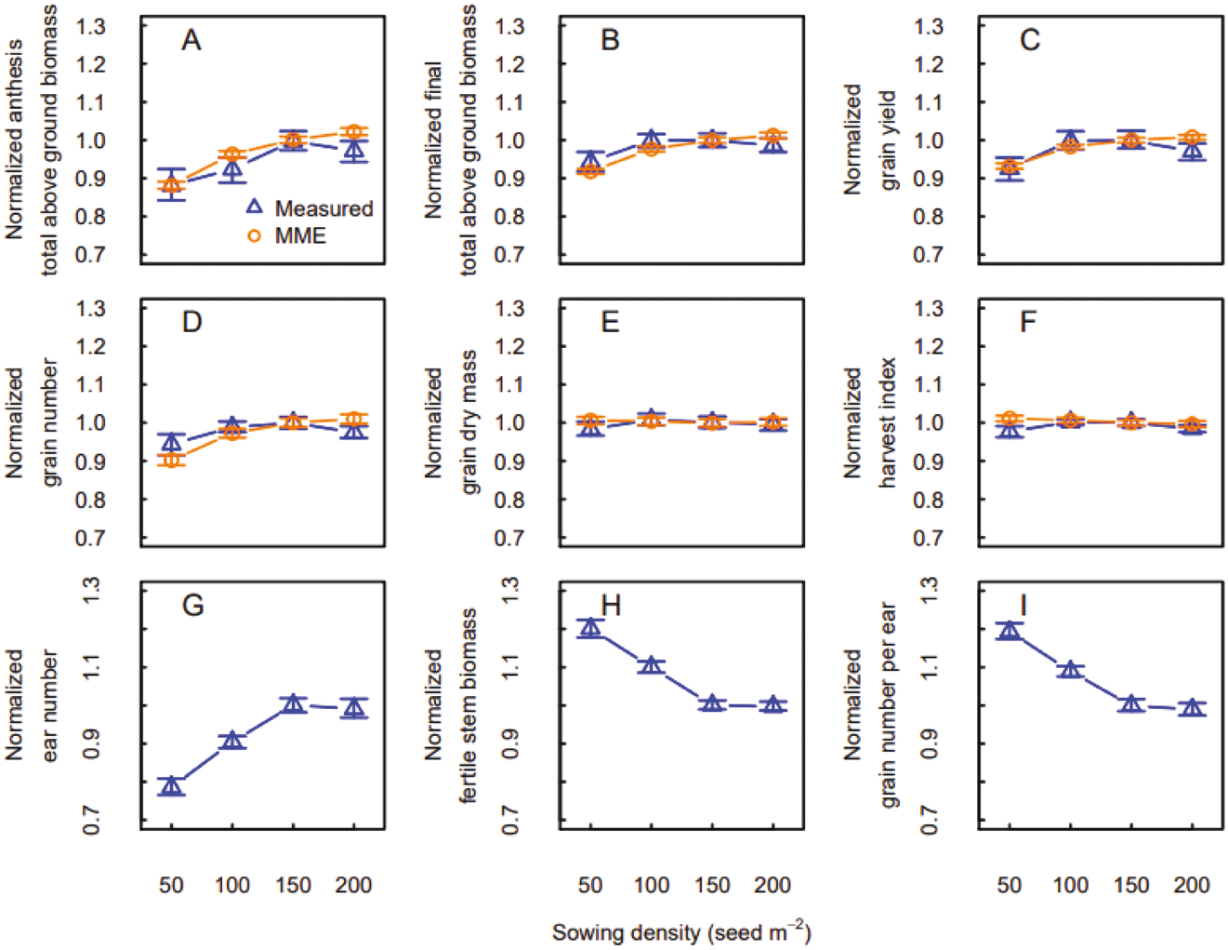
Measured (blue triangles) and simulated (orange circles) responses of total above ground biomass and yield components to sowing density for the winter wheat cultivar ‘Wakanui’ sown within the locally recommended sowing window (late March and April) for three consecutive years in Leeston (2012–2013 to 2014–2015), New Zealand. (A) Total above ground biomass at anthesis, (B) total above ground biomass at maturity, (C) grain yield, (D) grain number, (E) average grain dry weight, (F) harvest index, (G) ear number per m^2^, (H) fertile stem biomass, and (I) grain number per ear. Ear number, fertile stem biomass and grain number per ear are not simulated by the wheat crop growth models. Values were normalized using the mean of the measurements and simulations at 150 plants m^−2^ across years. Data are medians and error bars are 25%–75% quantiles for *n*=4 independent replicates (measurements) or the multi-model ensemble (simulations).

The MME simulations represented well the response to sowing density when the crop was sown at the locally recommended sowing date (Fig. 4). At very low sowing densities, total above ground biomass at anthesis and maturity (Supplementary Fig. S1), grain number, and grain yield all increased with increasing sowing density in both measurements and MME simulations. The increase reached a plateau at 100 or 150 seeds m^−2^ in the simulations, while in the measurements we observed a slight decrease between 150 and 200 seeds m^−2^. Conversely, average grain dry mass and harvest index showed little change with sowing density (Fig. 4E, F). The response of measured total biomass and grain yield to sowing density was similar to the response of ear number, while the number of grains per ear and the fertile stem biomass showed a ­decreasing response to sowing density followed by a stabilization around 150 seeds m^−2^.

**Fig. 5.**
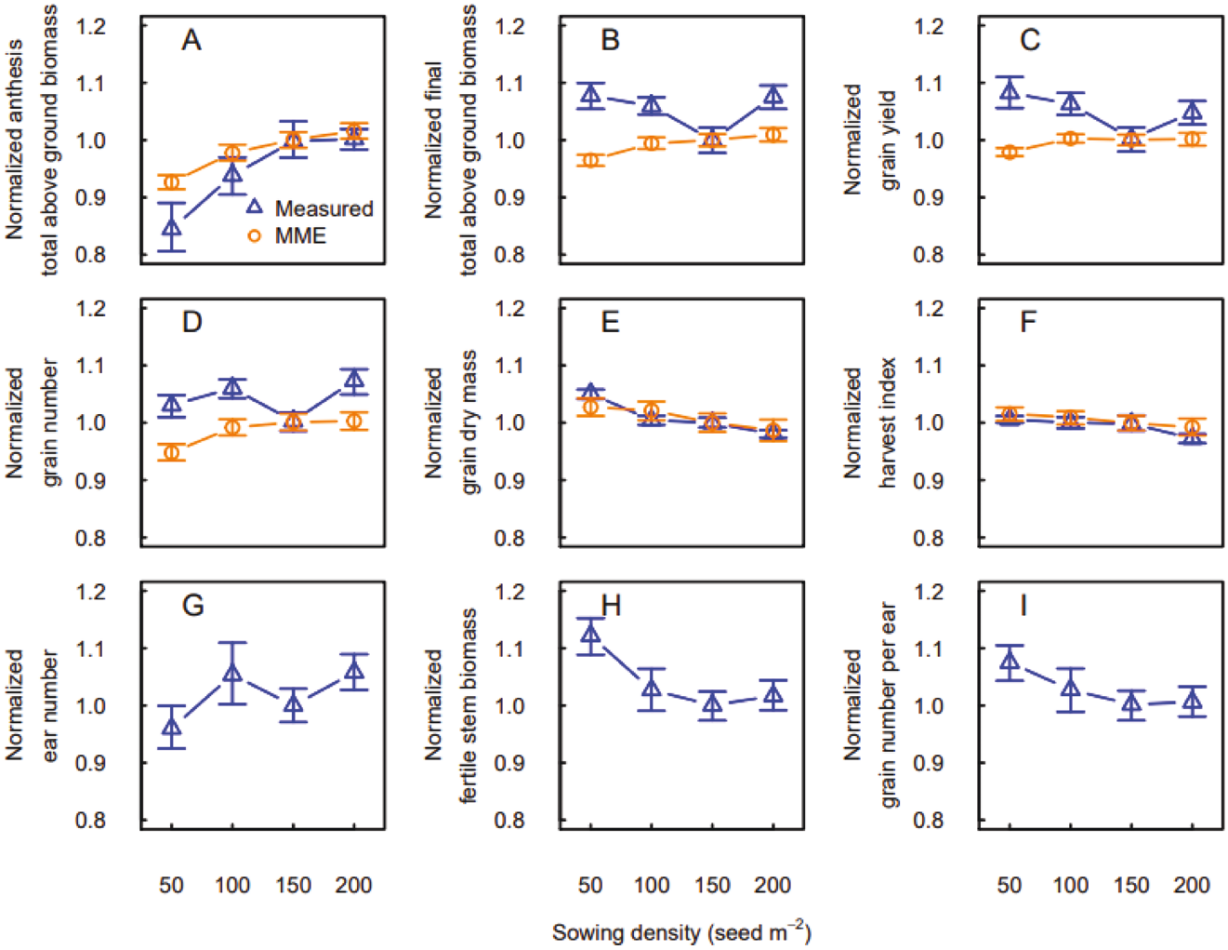
Measured (blue triangles) and simulated (orange circles) responses of total above ground biomass and yield components to sowing density for early sown (late February to early march) winter wheat crops in Leeston (2012–2013 to 2014–2015), New Zealand. (A) Total above ground biomass at anthesis, (B) total above ground biomass at maturity, (C) grain yield, (D) grain number, (E) average grain dry weight, (F) harvest index, (G) ear number per m^2^, (H) fertile stem biomass, and (I) grain number per ear. Ear number, fertile stem biomass, and grain number per ear are not simulated by the wheat crop growth models. Values were normalized using the mean of the measurements and simulations at 150 plants m^−2^ across years. Data are medians and error bars are 25%–75% quantiles for *n*=4 independent replicates (measurements) or the multi-model ensemble (simulations).

Early sowing changed the response of measured above ground biomass and grain yield to sowing density, and both decreased when sowing density increased from 50 to 150 seeds m^−2^. The measured fertile stem biomass and the grain number per ear showed a similar response. Conversely, the response of simulated total above ground biomass at anthesis and maturity, grain number, and grain yield did not change (Fig. 5). Measured grain number and ear number did not show a clear pattern. Interestingly, the response of the measured total above ground biomass at anthesis to sowing density was different from the one measured at maturity (Fig. 5A, B).

### Effect of sowing date

**Fig. 6.**
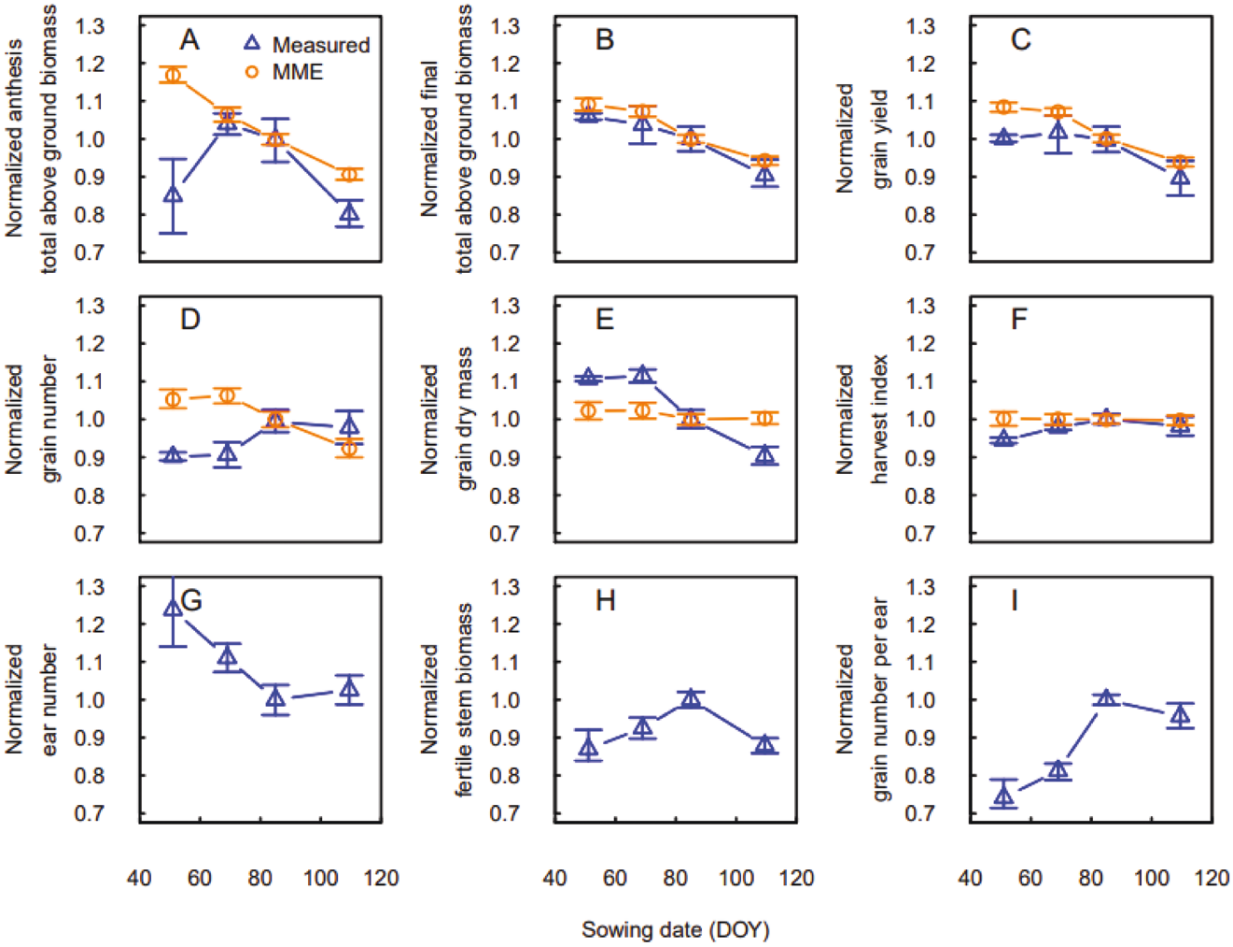
Measured (blue triangles) and simulated (orange circles) responses of total above ground biomass and yield components to sowing date for winter wheat crops sown at low density (50 plants m^−2^) in Leeston (2012–2013 to 2014–2015), New Zealand. (A) Total above ground biomass at anthesis, (B) total above ground biomass at maturity, (C) grain yield, (D) grain number, (E) average grain dry weight, (F) harvest index, (G) ear number per m^2^, (H) fertile stem biomass, and (I) grain number per ear. Ear number, fertile stem biomass, and grain number per ear are not simulated by the wheat crop growth models. Values were normalized using the mean of the measurements and simulations for the late March sowings. Data are medians and error bars are 25%–75% quantiles for *n*=4 independent replicates (measurements) or the multi-model ensemble (simulations).

For low sowing density (50 seeds m^−2^), under earlier sowing the final total above ground biomass and grain yield increased for both measurements and MME simulations (Fig. 6). February sowing had a negative effect on total above ground biomass at anthesis, and this was not captured by the MME. Grain number and average grain dry mass were poorly simulated by the MME.

Although the MME simulations gave a correct representation of the response of final total above ground biomass and grain yield to changes in sowing date, the underlying mechanism seemed to be wrong. While in measurements, the decrease in grain yield for later sowing was linked to the decrease in average grain dry mass, in the MME the decrease in yield was driven by a decrease in grain number, which was not present in the measurements.

**Fig. 7.**
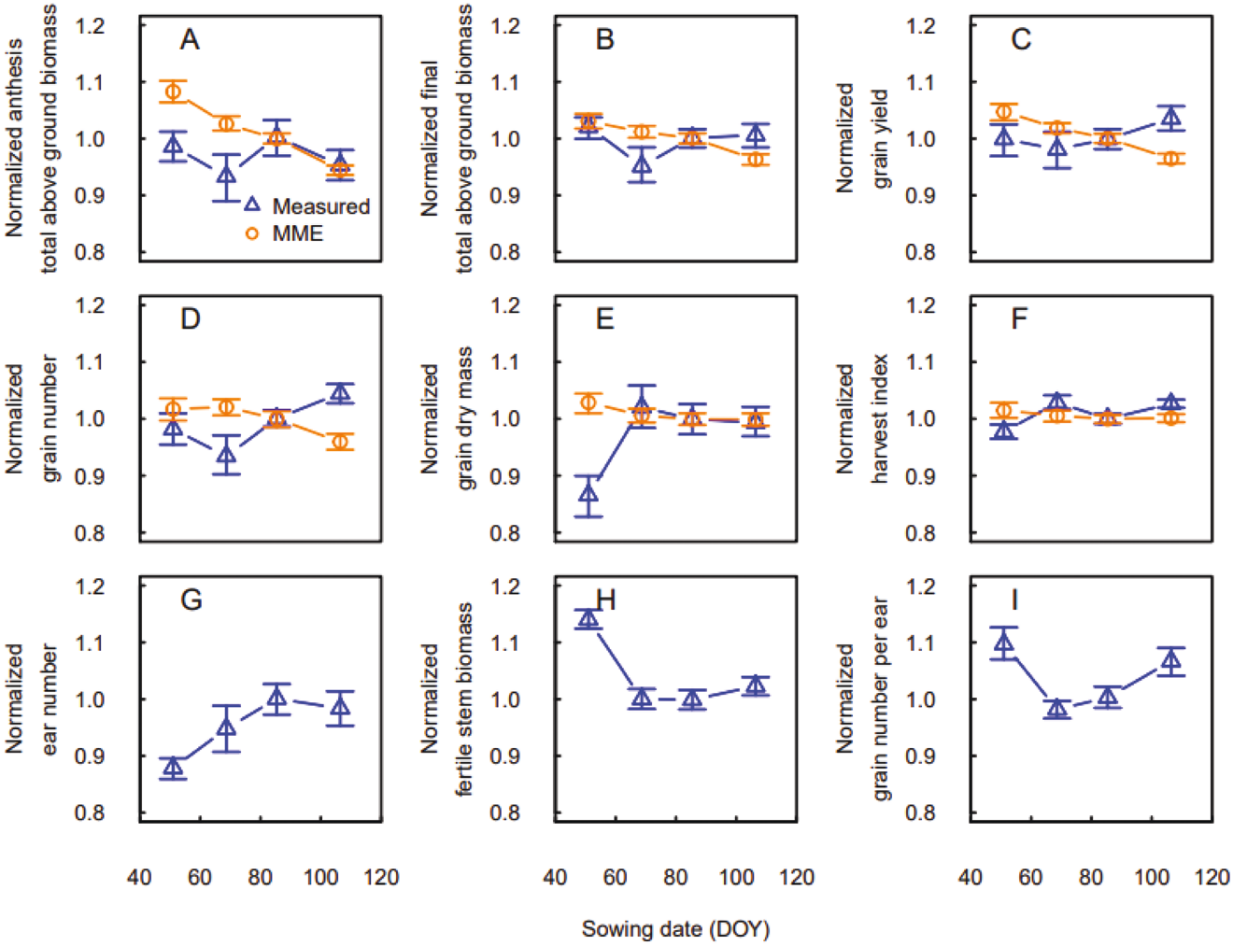
Measured (blue triangles) and simulated (orange circles) responses of total above ground biomass and yield components to sowing date for winter wheat crops sown at the locally recommended density (150 plants m^−2^) in Leeston (2012–2013 to 2014–2015) or Wakanui (2015–2016 and 2017–2018), New Zealand. (A) Total above ground biomass at anthesis, (B) total above ground biomass at maturity, (C) grain yield, (D) grain number, (E) average grain dry weight, (F) harvest index, (G) ear number per m^2^, (H) fertile stem biomass, and (I) grain number per ear. Ear number, fertile stem biomass and grain number per ear are not simulated by the wheat crop growth models. Values were normalized using the mean of the measurements and simulations for the late March sowings. Data are medians and error bars are 25%–75% quantiles for *n*=4 independent replicates (measurements) or the multi-model ensemble (simulations).

For locally recommended sowing density (150 seeds m^−2^), the effect of sowing date on final above ground biomass and grain yield initially showed a decrease between February and early March sowing and then an increase (Fig. 7). In this case, delaying sowing did not lead to a decrease in final total above ground biomass and grain yield, as shown by the MME simulation and observed at low sowing density (Fig. 6). The measurements showed that early sowing decreases ear number. The decrease of biomass and yield between February and early March sowing was related to the decrease of the fertile stem biomass and the grain number per ear.

### Effect of sowing date on in-season dynamics of crop growth

**Fig. 8.**
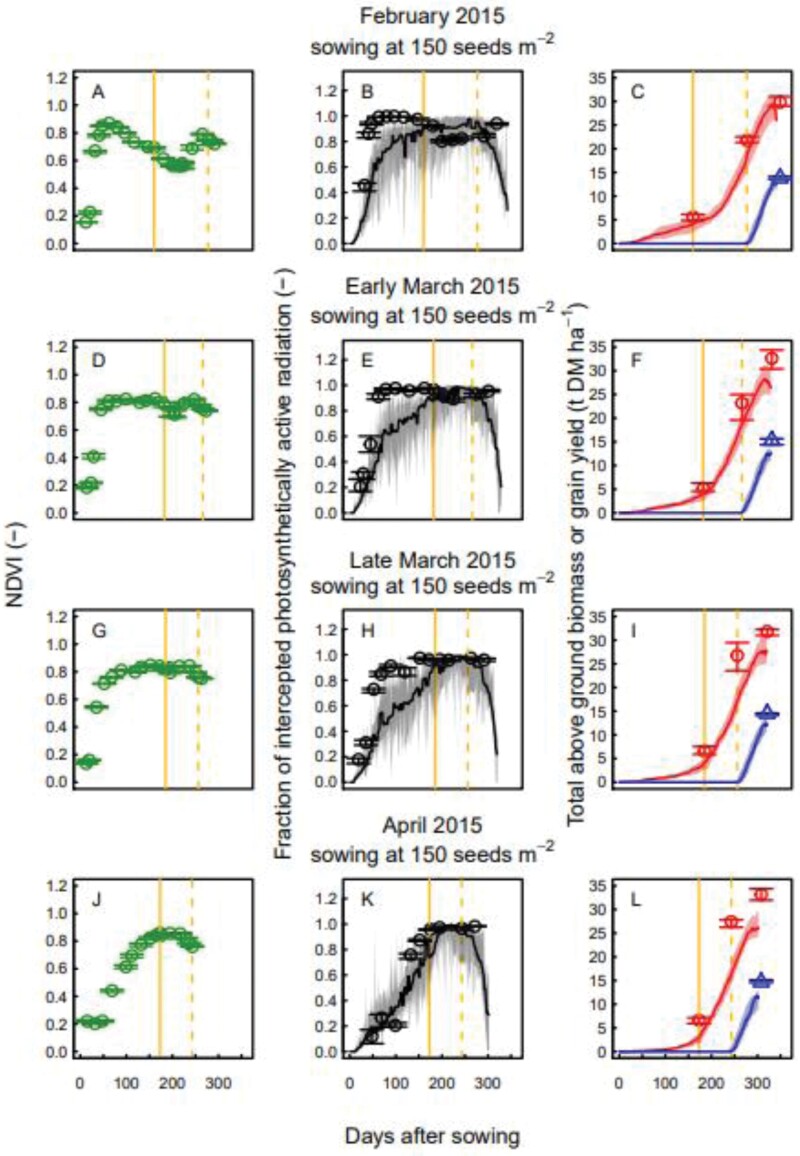
Measured normalized difference vegetation index (A, D, G, J), and measured and simulated fraction of intercepted PAR (B, E, H, K), and total above ground biomass (red) and grain yield (blue) (C, F, I, L) versus days after sowing for wheat crops sown on 20 February (A, B, C), 10 March (D, E, F), 20 March (G, H, I), and 9 April (J, K, L) 2015 at the locally recommended density (150 seeds m^−2^). Vertical yellow lines indicate the observed beginning of stem elongation (solid lines) and anthesis (dashed lines). Measured data (symbols) are medians for *n*=4 independent replicates and simulated data (lines) are medians for the multi-model ensemble. Error bars (measurements) and colour bandings (simulations) show 25%–75% quantiles.

For February, early March, and late March sowing, measured NDVI increased sharply after sowing, while April sowing resulted in a delayed increase (Fig. 8; Supplementary Fig. S2). In 2015 and for locally recommended sowing density, February sowing showed a clear decrease after the sharp increase, while for later sowings NDVI values remained high. In wheat sown in February, the decrease of NDVI continued up to the end of winter and then started increasing again. Compared with NDVI, measured values of FIPAR showed a smoother winter decrease. Nevertheless, February sown wheat showed a reduction of light interception. In 2014 and at low sowing density (50 seeds m^−2^) the decrease of NDVI and FIPAR during winter was also observed for March sowings, highlighting that the decrease could be observed in later sowings (Supplementary Fig. S2).

Comparing the MME simulations with measured values of FIPAR we identified two phases where the MME did not accurately represent the response of light interception. For February and March sowing, during early crop development, the MME underestimated the steep increase of FIPAR and total above ground biomass due to early vegetative growth. The MME simulations missed the decrease of light interception during late winter and overestimated FIPAR during this phase. This was partly compensated by the overestimation of FIPAR between stem elongation and anthesis, especially for crops sown in February. This compensation had a positive effect on the accuracy of the prediction of total above ground biomass and grain yield, which were close to the measured values for February sowing, while March and April sowing overestimated the final above ground biomass.

## Discussion

The MME simulations estimated the yield response to sowing rate well for the locally recommended sowing date, but the simulations were less accurate for earlier sowing dates (Fig. 1). In general, increasing sowing rate increases the number of plants per unit area, but at high densities competition for resources (light, nutrients, and water) can limit leaf and tiller production. Several studies have documented yield increases and saturation of yield response with increased sowing rate (Spink *et al.*, 2000; Postma *et al.*, 2020) and have described limited biomass growth and increased tiller mortality due to excess competition. Competition for light is the primary limiting factor, as it becomes important even when nutrients and water are not limiting (Postma *et al.*, 2020). Indeed, increasing sowing density expands shading for part of the foliage of neighbouring plants, reducing plant photosynthesis (Postma *et al.*, 2020). Thus, increasing sowing rate should increase final above ground biomass and grain yield up to an optimum threshold (Fig. 4), but early sowing, by promoting early vegetative growth and competition among tillers, shifts this threshold to lower sowing rates (Fig. 5). This offsets the expected positive effect of increased sowing rate on yield for early sowing dates.

Early sowing increases the risk of diseases and pests, but sowing date also has an important effect on tillering and tiller mortality. Early tillers have several growth advantages, including higher leaf area, greater number of grains, higher grain weight, and an enhanced resistance to stresses (Tilley *et al.*, 2019). Several studies have shown earlier sowing dates can increase grain yield (Green and Ivins, 1985; Sun *et al.*, 2013; Gandjaeva, 2019; Tahir *et al.*, 2019). However, sowing wheat too early can also cause excessive tillering, increase tiller mortality, and result in lower grain yield (Thiry *et al.*, 2002). In our experiments, for low sowing density (50 seeds m^−2^), final total above ground biomass and grain yield increased with earlier sowing (Fig. 6). The response was related to an increased number of spikes with earlier sowing, a corresponding increase in grain weight, while the number of grains per ear decreased. The MME simulations represented the response on yield well, although they missed the underlying mechanism since the increase in simulated yield was driven by an increase in grain number with earlier sowing, and not by an increase of ear number and grain dry mass as in measurements. For the currently used sowing rate (150 seeds m^−2^), early sowing increased tillering, and thus increased competition and tiller mortality (Garcia del Moral and Garcia del Moral, 1995; Spink *et al.*, 2000). As a result, and contrary to what was observed at low sowing rates, the measured ear number decreased with earlier sowing (Fig. 7).

The NDVI and FIPAR measurements clearly showed the effect of senescence after an initial rapid vegetative growth. Overall, in early sown crops FIPAR showed a smaller winter decline than NDVI. FIPAR represents the canopy area involved in photosynthesis, while NDVI combines canopy area and greenness, and thus represents canopy health and photosynthetic potential of the canopy. For the February 2015 and February and March 2014 sowings, we observed a decrease in measured NDVI and FIPAR after a rapid increase. The decrease is observed with the February sowing in 2015 for a sowing rate of 150 seeds m^−2^ and with February sowing and March sowing in 2014 for a sowing rate of 50 seeds m^−2^. Since senescence occurs when there is high competition for resources between tillers (Hecht *et al*, 2019), we expected to observe lower levels of senescence at lower sowing rates. However, the data showed a stronger decrease of NDVI and FIPAR for later sowing dates for the treatments with lower sowing rates. To explain this, we need to consider the interannual variability of temperature, which also affects tiller production and leaf growth. A higher temperature means that leaf initiation occurs earlier and leaf development is faster. Indeed, 2014 was characterized by a higher average temperature from April to September. This may have favoured tiller and leaf production and increased tiller competition.

Underestimation of initial vegetative growth for February and March sowing dates (Fig. 8; Supplementary Fig. S2) explains why in some years the MME did not reach the measured biomass at anthesis (Fig. 2). Problems in representing initial vegetative growth and senescence for early sowing dates may also have affected model calibration. The MME was not able to represent the decrease in FIPAR after the initial rapid growth that was measured at early sowing dates in the calibration year (2014–2015, Supplementary Fig. S2). Therefore, the MME tended to overestimate yield for early sowing dates. Since the model calibration was performed using all 2014–2015 sowing conditions, this may have led to a parameterization that compensated for the overestimation of early sowing, resulting in an underestimation of grain yield for the recommended sowing dates.

Only 13 of the 29 models reported the FIPAR, and of these, only three (AE, HE, and SQ) simulated a decrease in FIPAR after the rapid increase with the February sowing in 2015 and only two (HE, SQ) with the February sowing in 2014, while none of the models simulated the decrease measured with the March sowing in 2014. Interestingly, the models that were more successful in simulating the FIPAR response represent different modelling approaches, SQ being a very sophisticated model that represents tillering, grain number, and size explicitly, while HE is based on a one-leaf and one-grain approach. Furthermore, the models that captured the decrease in FIPAR did not show better performance in simulating yield (Supplementary Tables S2, S3, S4). This showed that the ability to simulate the physiological response did not guarantee a better ability to simulate the target variable (grain yield). While some models may include some of the processes responsible for the observed responses, none of them has been able to represent the full complexity of the processes involved. We suggest that improving the representation of tillering and tiller competition in crop growth models may increase model accuracy when simulating high-tillage varieties.

The comparison of the MME and experimental data highlighted that the MME was not able to correctly represent the response of yield to increasing sowing rate at early sowing dates and the response to sowing date at the currently recommended sowing rate. Some of the models showed better performance than the MME in predicting grain yield at an early sowing date, but none was more accurate than the MME at a low sowing rate (Supplementary Tables S3, S4).

### Conclusion

Although previous studies have shown that MMEs were effective in reducing uncertainty in predicting yield (Rötter *et al.*, 2011; Asseng *et al.*, 2013; Bassu *et al.*, 2014; Li *et al.*, 2015; Fleisher *et al.*, 2017), soil water, biomass (Rötter *et al.*, 2012), and other crop growth variables (Martre *et al.*, 2015), our results indicate that this might not apply to all growing conditions, such as early sowing and low sowing rates. Some underlying mechanisms, such as tillering, may not be well represented in the MME and therefore in most crop models. This is important to consider when using a MME approach or single crop models to evaluate the impact of a specific management adaptation (such as sowing date and sowing density) in wheat cropping systems to climate change projections. As the adaptation of sowing date is considered one of the most important measures for climate change adaptation, it is important to be aware of the possible limitations of model accuracy.

In this study, models were used to simulate crop growth in a high-yielding environment where sowing conditions were modified to achieve even higher yields. Representing physiological responses under these extreme conditions is a huge challenge for wheat crop models. In addition, some responses may have been affected by pest and diseases, which are not represented by the models, making modelling even more challenging. Although the models have encountered difficulties in representing extreme conditions, these results are useful in guiding the development of new wheat genotypes. The response of a wheat idiotype with a very erect canopy, higher potential spike population, high lodging and disease resistance, later anthesis, and a longer grain filling period would likely be in agreement with the yield increase predicted by current models for early sowing. Therefore, understanding why models fail to accurately predict a measured response may point to avenues for genotype improvement and guide breeding efforts.

## Supplementary data

The following supplementary data are available at *JXB* online.

Fig. S1. Measured and simulated fraction of intercepted PAR, and total above ground biomass and grain yield versus days after sowing for wheat crops sown 16 April 2013 at the low and at the locally recommended density.

Fig. S2. Measured normalized difference vegetation index, measured and simulated fraction of intercepted PAR, and total above ground biomass and grain yield versus days after sowing for wheat crops sown on 20 February, 10 March, 26 March, and 23 April 2014 at the locally recommended density (150 seeds m^−2^).

Table S1. List of the 29 wheat crop models used in the AgMIP Wheat Phase 4 study

Table S2. Statistical evaluation of model error and interannual variability of measured and simulated grain yield, considering wheat sown at the locally recommended sowing date and plant density.

Table S3. Statistical evaluation of model error in grain yield, considering wheat sown at the locally recommended plant density vs low sowing density.

Table S4. Statistical evaluation of model error in grain yield, considering wheat sown at the locally recommended date versus early sowing.

Protocol S1. Crop model performance and evaluation metrics.

erac221_suppl_Supplementary_Materials

## Data Availability

The data that support the findings of this study are openly available in Harvard Dataverse at https://doi.org/10.7910/DVN/XA4VA2.
