# Supplementary material for: Simulation of winter wheat response to variable sowing dates and densities in a high-yielding environment
Source: J Exp Bot. 2022 Jun 21;73(16):5715–29. doi: 10.1093/jxb/erac221 (PMC9467659; doi:10.1093/jxb/erac221)
Supplement: erac221_suppl_Supplementary_Materials [file erac221_suppl_supplementary_materials.pdf]

Supplementary Information

Table S1. List of the 29 wheat crop models used in the AgMIP Wheat Phase 4 study.

Code	Name (version)	Reference	Documentation
AE	APSIM Next Gen	Holzworth et al. (2014), Brown et al. (2018)	https://apsimnextgeneration.netlify.app/modeldocumentation/
AQ	AQUACROP (V.4.0)	Steduto et al. (2009)	http://www.fao.org/nr/water/aquacrop.html
AW	APSIM-Wheat (V.7.3)	Keating et al. (2003)	https://www.apsim.info/documentation/model-documentation/crop-module-documentation/wheat/
CS	CropSyst (V.3.04.08)	Stockle et al. (2003)	http://modeling.bsyse.wsu.edu/CS_Suite_4/CropSyst/index.html
D1<br>D3<br>D4	DSSAT-CERES-Wheat (V.4.7.1.001)	Ritchie et al. (1985); Hoogenboom and White (2003); Jones et al. (2003)	http://dssat.net/
DN	DSSAT-NWheat (V4.7.5.0)	Asseng (2004); Kassie et al. (2016)	http://dssat.net/
DR	DSSAT-CROPSIM (V4.5.1.013)	Hunt and Pararajasingham (1995); Jones et al. (2003)	http://dssat.net/
DS	DAISY (V.5.24)	Hansen et al. (1991); Hansen et al. (2012)	http://daisy.ku.dk
HE	HERMES (V.4.26)	Kersebaum (2007); Kersebaum (2011)	http://www.zalf.de/en/forschung/institute/lsa/forschung/oekomod/hermes
L5	SIMPLACE<Lintul-5a>	Gaiser et al. (2013); Webber et al. (2016)	http://www.simplace.net/doc/
L6	SIMPLACE<Lintul-5b>	Angulo et al. (2013)	http://www.simplace.net/doc/
LI	LINTUL4 (V.1)	Spitters and Schapendonk (1990); Shibu et al. (2010)	http://models.pps.wur.nl/node/950
MC	MCWLA-Wheat (V.2.0)	Tao et al. (2009); Tao et al. (2009); Tao and Zhang (2010); Tao and Zhang (2013)	Request from taofl@igsnrr.ac.cn
MO	MONICA (V.1.0)	Nendel et al. (2011)	http://monica.agrosystem-models.com
NC	Expert-N (V3.0.10) – CERES (V2.0)	Ritchie et al. (1987); Stenger et al. (1999); Priesack et al. (2006); Biernath et al. (2011)	https://expert-n.uni-hohenheim.de/

NG	Expert-N (V3.0.10) – GECROS (V1.0)	Stenger et al. (1999); Biernath et al. (2011)	https://expert-n.uni-hohenheim.de/
NP	Expert-N (V3.0.10) – SPASS (2.0)	Stenger et al. (1999); Wang and Engel (2000); Yin and van Laar (2005); Priesack et al. (2006); Biernath et al. (2011)	https://expert-n.uni-hohenheim.de/
NS	Expert-N (V3.0.10) – SUCROS (V2)	Goudriaan and Van Laar (1994); Stenger et al. (1999); Priesack et al. (2006); Biernath et al. (2011)	https://expert-n.uni-hohenheim.de/
PG	pyGECROS (v2019)	Yin and van Laar (2005)	https://github.com/andres-berger/pygecros
S2	Sirius (V2014)	Jamieson et al. (1998); Jamieson and Semenov (2000); Stratonovitch and Semenov (2015); Senapati et al. (2019)	https://sites.google.com/view/sirius-wheat/; https://doi.org.10.5281/zenodo.4572624
SA	SALUS (V.1.0)	Senthilkumar et al., (2009); Basso et al. (2010)	http://salusmodel.glg.msu.edu
SP	SIMPLACE<Lintul-2>	Angulo et al. (2013)	http://www.simplace.net/doc/
SQ	*SiriusQuality* (V3.0)	Martre et al. (2006); Ferrise et al. (2010); He et al. (2010); Maiorano et al. (2017)	http://www1.clermont.inra.fr/siriusquality
SS	SSM-iCrops (v1)	Soltani et al. (2013)	Request from afshin.soltani@gmail.com
WG	WheatGrow (V3.1)	Cao and Moss (1997); Yan et al. (2001); Cao et al. (2002); Li et al. (2002); Hu et al. (2004); Pan et al. (2006); Pan et al. (2007)	Request from yanzhu@njau.edu.cn
WO	WOFOST (V.7.1)	Boogaard el al. (1998)	http://www.wofost.wur.nl
WU	WOFOST_UGOE (V.7.2)	Boogaard et al. (2014); Pirttioja et al. (2015); de Wit et al (2019)	http://www.wofost.wur.nl

Table S2. Relative root mean squared relative error (RRMSE), decomposition of mean squared error in lack of correlations (LC), squared bias (SB), and non-unity (NU) slope, Nash-Sutcliffe modelling efficiency (EF), and measured and simulated interannual variability for yield. Data are from the wheat cultivar 'Wakanui' sown at the locally recommended sowing date and plant density for six consecutive years in Leeston or Wakanui, New Zealand.

Measurements or models	RRMSE (%)	LC	SB	NU	EF	Interannual variability (%CV)
Measurements	-	-	-	-	-	10.87
NC	6.10	0.60	0.01	0.00	0.65	9.43
WO	10.27	1.53	0.11	0.10	0.02	6.81
MME	10.33	1.61	0.01	0.14	0.01	6.4
L5	10.60	1.65	0.10	0.11	-0.05	5.62
LI	10.60	1.31	0.00	0.54	-0.05	11.5
NP	12.11	1.77	0.02	0.63	-0.37	7.23
D1	12.25	1.73	0.39	0.36	-0.40	6.34
MO	12.72	1.74	0.18	0.75	-0.51	8.85
AW	12.77	1.69	0.61	0.39	-0.52	7.09
L6	13.01	1.66	0.00	1.14	-0.58	5.96
DR	13.07	1.77	0.19	0.87	-0.59	8.36
S2	13.20	1.28	1.60	0.00	-0.62	4.93
DN	13.37	1.72	1.17	0.07	-0.67	4.37
SP	13.76	1.77	0.12	1.24	-0.76	8.29
WU	13.94	1.69	1.25	0.28	-0.81	7.35
SQ	14.05	1.69	0.83	0.74	-0.84	10.03
CS	15.28	1.33	0.07	2.46	-1.17	17.87
HE	15.90	1.54	0.42	2.21	-1.36	15.28
NS	16.20	1.77	0.80	1.77	-1.44	11.11
WG	16.36	1.52	2.64	0.26	-1.49	7.39
AQ	16.83	1.3048	2.3527	1.0236	-1.6402	2.39
NG	16.99	1.72	0.00	3.05	-1.69	12.3
MC	17.97	1.69	0.00	3.64	-2.01	18.02
D4	17.99	1.65	2.47	1.23	-2.02	10.63
SA	19.54	1.70	0.25	4.35	-2.56	14.28
PG	22.94	1.77	5.10	1.82	-3.90	13.78
SS	25.90	1.67	0.02	9.39	-5.25	22.16
DS	31.13	1.73	8.49	5.79	-8.03	23.27
AE	38.37	0.80	23.15	0.37	-12.72	20.81
D3	38.88	1.40	17.86	5.70	-13.08	21.65

Table S3. Relative root mean squared relative error (RRMSE), decomposition of mean squared error in lack of correlations (LC), squared bias (SB), and non-unity (NU) slope, Nash-Sutcliffe modelling efficiency (EF) for yield, considering locally recommended and low sowing density. Models are ordered by increasing values of RRMSE of global dataset (see Table S1). Data are from the wheat cultivar 'Wakanui' and includes all sowing dates for three consecutive years in Leeston, New Zealand.

	Locally recommended sowing density (150 seeds m^{-2})					Low sowing density (50 seeds m^{-2})				
Models	RRMSE	*LC*	*SB*	NU	*EF*	RRMSE	*LC*	*SB*	NU	*EF*
NC	8.65	0.97	0.18	0.06	0.37	7.61	0.29	0.51	0.06	-0.05
WO	9.59	1.45	0.03	0.00	0.22	6.60	0.59	0.03	0.02	0.21
MME	11.07	1.747	0.10	0.13	-0.04	5.46	0.39	0.04	0.01	0.46
L5	11.40	1.88	0.00	0.21	-0.10	6.30	0.46	0.13	0.00	0.28
LI	10.51	1.40	0.07	0.31	0.07	10.06	0.80	0.57	0.13	-0.83
NP	12.95	1.90	0.07	0.73	-0.42	10.27	0.77	0.63	0.17	-0.91
D1	13.15	1.89	0.31	0.59	-0.46	7.86	0.67	0.05	0.20	-0.12
MO	14.49	1.90	0.01	1.47	-0.78	8.87	0.44	0.06	0.67	-0.42
AW	15.22	1.86	1.37	0.49	-0.96	11.39	0.58	0.34	1.01	-1.35
L6	13.73	1.68	0.00	1.36	-0.60	8.99	0.65	0.55	0.00	-0.46
DR	15.00	1.73	0.53	1.37	-0.90	16.13	0.49	2.37	1.01	-3.71
S2	14.40	1.19	2.11	0.04	-0.75	7.33	0.43	0.33	0.04	0.03
DN	12.18	1.74	0.65	0.00	-0.25	13.48	0.80	1.89	0.01	-2.29
SP	14.35	1.90	0.63	0.79	-0.74	10.13	0.70	0.72	0.11	-0.86
WU	13.29	1.75	1.00	0.09	-0.49	8.95	0.51	0.66	0.02	-0.45
SQ	15.70	1.88	0.09	2.00	-1.09	9.63	0.33	0.85	0.20	-0.68
CS	13.89	1.39	0.21	1.51	-0.63	15.31	0.28	1.30	1.90	-3.24
HE	17.99	1.69	1.39	2.12	-1.74	13.43	0.42	0.99	1.27	-2.27
NS	16.09	1.89	0.02	2.25	-1.19	9.65	0.81	0.16	0.41	-0.69
WG	17.35	1.62	3.08	0.14	-1.54	10.60	0.73	0.62	0.32	-1.03
AQ	18.86	1.315	3.243	1.166	-2.007	12.57	0.82	1.47	0.06	-1.86
NG	19.69	1.84	0.74	3.66	-2.28	39.33	0.51	19.13	3.33	-27.00
MC	18.30	1.86	0.23	3.30	-1.83	13.45	0.50	1.22	0.96	-2.27
D4	19.61	1.77	3.60	0.82	-2.25	14.43	0.57	0.30	2.22	-2.77
SA	18.93	1.90	0.89	2.99	-2.03	10.12	0.77	0.12	0.63	-0.85
PG	21.73	1.83	1.58	4.20	-3.00	10.08	0.63	0.01	0.88	-0.84
SS	29.08	1.90	2.06	9.66	-6.15	14.47	0.68	0.03	2.40	-2.79
DS	31.42	1.80	8.27	5.81	-7.35	16.06	0.55	0.97	2.31	-3.67
AE	35.96	1.06	19.23	0.53	-9.94	28.63	0.73	10.15	1.29	-13.83
D3	37.76	1.12	14.67	7.16	-11.06	16.73	0.81	2.73	0.61	-4.07

Table S4. Relative root mean squared relative error (RRMSE), decomposition of mean squared error in lack of correlations (LC), squared bias (SB), and non-unity (NU) slope, Nash-Sutcliffe modelling efficiency (EF) for yield, considering locally recommended and early sowing date. Simulated data are for 29 wheat crop growth models and the multi-model ensemble (MME) median. Models are ordered by increasing values of RRMSE of global dataset (see Table S1). Data are from the wheat cultivar 'Wakanui' and includes all plant densities for six consecutive years in Leeston or Wakanui, New Zealand.

	Locally recommended sowing date (Late March and April)					Early sowing date (February and early March)				
Models	RRMSE	*LC*	*SB*	NU	*EF*	RRMSE	*LC*	*SB*	NU	*EF*
NC	6.89	0.67	0.08	0.00	0.57	11.62	0.43	1.41	0.15	-3.62
WO	9.75	1.21	0.13	0.16	0.15	6.44	0.42	0.10	0.09	-0.42
MME	8.33	0.96	0.04	0.10	0.38	12.31	0.32	1.58	0.32	-4.18
L5	8.83	0.90	0.14	0.19	0.30	11.12	0.37	1.11	0.34	-3.23
LI	9.25	0.74	0.20	0.41	0.23	9.44	0.37	0.51	0.43	-2.05
NP	13.37	1.62	0.33	0.86	-0.60	14.20	0.40	2.43	0.13	-5.90
D1	7.73	0.70	0.22	0.02	0.46	7.23	0.43	0.00	0.34	-0.79
MO	10.31	1.46	0.02	0.20	0.05	19.34	0.28	4.68	0.53	-11.78
AW	15.41	1.47	0.70	1.57	-1.13	20.32	0.32	4.32	1.42	-13.11
L6	10.11	1.56	0.02	0.02	0.08	10.32	0.42	0.88	0.26	-2.64
DR	13.41	1.24	1.20	0.40	-0.62	23.41	0.40	3.22	4.42	-17.73
S2	10.60	0.77	0.91	0.09	-0.01	15.11	0.42	2.36	0.57	-6.81
DN	14.97	1.75	1.61	0.17	-1.01	9.50	0.31	0.27	0.74	-2.09
SP	11.52	1.65	0.00	0.44	-0.19	15.45	0.38	2.92	0.21	-7.15
WU	12.75	0.91	1.38	0.27	-0.46	7.05	0.34	0.19	0.19	-0.70
SQ	6.24	0.56	0.05	0.00	0.65	18.61	0.35	4.46	0.27	-10.84
CS	14.69	0.31	1.50	1.59	-0.94	7.52	0.42	0.09	0.32	-0.94
HE	9.32	0.95	0.16	0.26	0.22	26.62	0.36	8.85	1.19	-23.23
NS	12.96	1.63	0.13	0.89	-0.51	16.89	0.36	2.58	1.24	-8.75
WG	10.40	0.63	1.04	0.03	0.03	17.95	0.29	2.73	1.72	-10.02
AQ	17.63	1.72	2.46	0.72	-1.789	21.49	0.33	5.64	0.80	-14.784
NG	27.70	1.64	3.72	6.71	-5.89	20.56	0.25	1.00	4.95	-13.45
MC	9.14	1.18	0.00	0.13	0.25	24.28	0.38	5.90	2.37	-19.16
D4	12.07	1.06	0.01	1.23	-0.31	20.50	0.32	4.07	1.77	-13.36
SA	21.12	1.40	0.23	5.39	-3.00	15.06	0.35	1.54	1.44	-6.75
PG	14.47	1.73	0.69	0.88	-0.88	17.94	0.41	3.63	0.69	-10.00
SS	26.76	1.16	3.97	6.15	-5.43	20.00	0.39	4.00	1.48	-12.68
DS	19.44	1.35	1.78	2.82	-2.39	23.88	0.35	5.30	2.72	-18.50
AE	32.52	1.22	15.12	0.31	-8.49	28.56	0.36	10.02	1.58	-26.87
D3	22.71	1.56	4.86	1.71	-3.63	13.77	0.35	2.42	0.02	-5.48

Figure S1: Measured and simulated fraction of intercepted PAR (A and C), and total above ground biomass and grain yield (B and D) versus days after sowing for wheat crops sown 16 April 2013 at the low sowing density (A and B) and at the locally recommended density (C and D). Vertical yellow lines indicate the observed anthesis (solid lines). Measured data (symbols) are medians for *n* = 4 independent replicates and simulated data (lines) are medians for the MME. Error bars (measurements) and color bandings (simulations) show 25% - 75% quantiles.

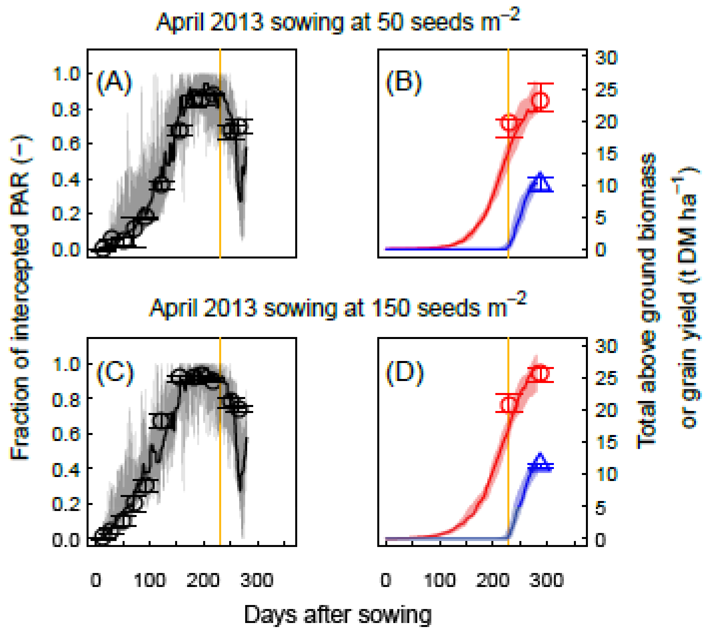

Figure S2: Measured normalized difference vegetation index (A,D,G, and J), and measured and simulated fraction of intercepted PAR (B,E,H, and K), and total above ground biomass and grain yield (C,F,I and L) versus days after sowing for wheat crops sown on 20 February (A, B, and C), 10 March (D, E, and F), 26 March (G, H, and I), and 23 April (J, K, and L) 2014 at the locally recommended density (150 seeds m^{-2}). Vertical yellow lines indicate the observed beginning of stem elongation (solid lines), and anthesis (dashed lines). Measured data (symbols) are medians for n = 4 independent replicates and simulated data (lines) are medians for the MME. Error bars (measurements) and color bandings (simulations) show 25% - 75% quantiles.

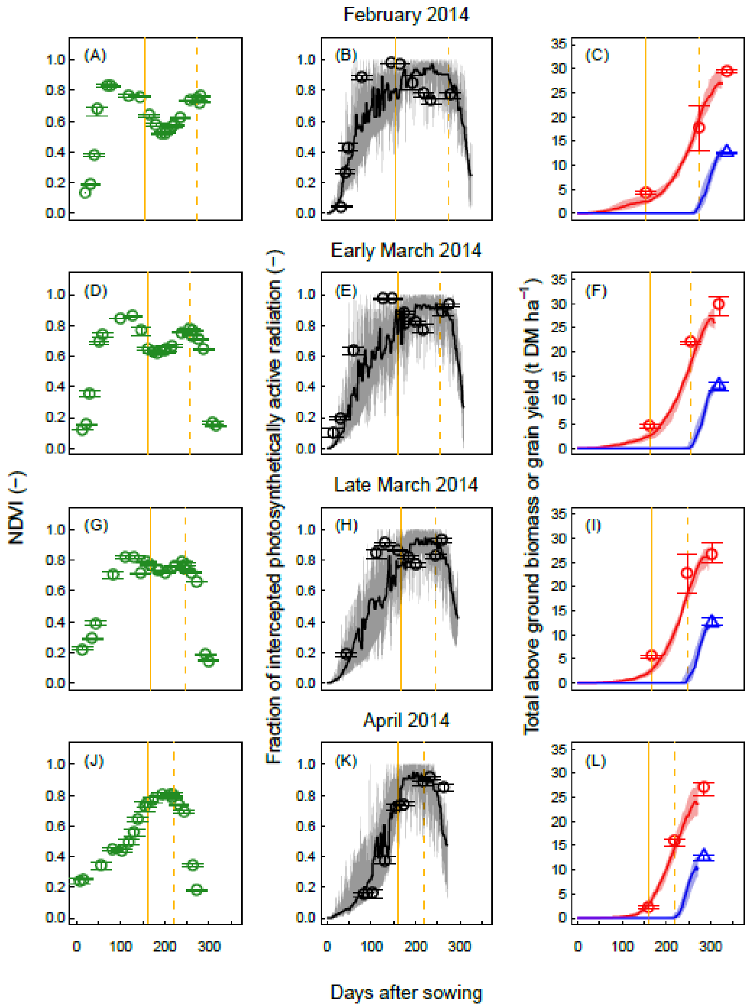

References

Angulo C, Rötter R, Lock R, Enders A, Fronzek S, Ewert F 2013. Implication of crop model calibration strategies for assessing regional impacts of climate change in Europe. Agricultural and Forest Meteorology 170: 32-46

Asseng S 2004. Wheat Crop Systems: A Simulation Analysis. CSIRO Publishing, Melbourne, Australia

Basso B, Cammarano D, Troccoli A, Chen D, Ritchie J 2010. Long-term wheat response to nitrogen in a rainfed Mediterranean environment: Field data and simulation analysis. European Journal of Agronomy 33: 132-138

Biernath C, Gayler S, Bittner S, Klein C, Hogy P, Fangmeier A, Priesack E 2011. Evaluating the ability of four crop models to predict different environmental impacts on spring wheat grown in open-top chambers. European Journal of Agronomy 35: 71-82

Boogaard HL, van Diepen CA, Rötter RP, Cabrera JMCA, van Laar HH 1998. WOFOST 7.1. User's guide for the WOFOST 7.1 crop growth simulation model and WOFOST Control Center 1.5. 52, DLO Winand Staring Centre, Wageningen

Boogaard HL, de Wit AJW, te Roller J, van Diepen CA 2014. User's guide for the WOFOST Control Centre 2.1 and WOFOST 7.1.7 crop growth simulation model. Alterra Wageningen University & Research Centre, Wageningen (2014), p. 133. http://www.wur.nl/web/file?uuid=5c0873c3-8c07-4ddf-85a3-dd98bdb38781&owner=b875561e-c6d9-442d-b599-58e9d13cb80d

Brown H, Huth N, Holzworth D 2018. Crop model improvement in APSIM: Using wheat as a case study. Eur J Agron 100: 141-150

Cao W, Liu T, Luo W, Wang S, Pan J, Guo W 2002. Simulating organic growth in wheat based on the organ-weight fraction concept. Plant Production Science 5: 248-256

Cao W, Moss DN (1997) Modelling phasic development in wheat: a conceptual integration of physiological components. Journal of Agricultural Science 129: 163-172

Ferrise R, Triossi A, Stratonovitch P, Bindi M, Martre P 2010. Sowing date and nitrogen fertilisation effects on dry matter and nitrogen dynamics for durum wheat: An experimental and simulation study. Field Crops Res 117: 245-257

Gaiser T, Perkons U, Küpper PM, Kautz T, Uteau-Puschmann D, Ewert F, Enders A, Krauss G 2013. Modeling biopore effects on root growth and biomass production on soils with pronounced sub-soil clay accumulation. Ecological modelling 256: 6-15

Goudriaan J, Van Laar HH, eds (1994) Modelling Potential Crop Growth Processes. Textbook With Exercises. Kluwer Academic Publishers, Dordrecht, The Netherlands

Hansen S, Abrahamsen P, Petersen CT, Styczen M 2012. DAISY: model use, calibration, and validation. Transaction of the ASABE 55: 1317-1335

Hansen S, Jensen H, Nielsen N, Svendsen H 1991. Simulation of nitrogen dynamics and biomass production in winter-wheat using the Danish simulation model DAISY. Fertilizer Research 27: 245-259

He J, Stratonovitch P, Allard V, Semenov MA, Martre P 2010. Global sensitivity analysis of the process-based wheat simulation model SiriusQuality1 identifies key genotypic parameters and unravels parameters interactions. Procedia - Social and Behavioral Sciences 2: 7676-7677

Holzworth DP, Huth NI, deVoil PG, *et al.* 2014. APSIM – Evolution towards a new generation of agricultural systems simulation. *Environmental Modelling & Software* **62:** 327-350.

Hoogenboom G, White J 2003. Improving physiological assumptions of simulation models by using gene-based approaches. Agronomy Journal 95: 82-89

Hu J, Cao W, Zhang J, Jiang D, Feng J 2004. Quantifying responses of winter wheat physiological processes to soil water stress for use in growth simulation modeling. Pedosphere 14: 509-518

Hunt LA, Pararajasingham S 1995. CROPSIM-wheat - a model describing the growth and development of wheat. Canadian Journal of Plant Science 75: 619-632

Jamieson P, Semenov M 2000. Modelling nitrogen uptake and redistribution in wheat. Field Crops Research 68: 21-29

Jamieson P, Semenov M, Brooking I, Francis G 1998. Sirius: a mechanistic model of wheat response to environmental variation. European Journal of Agronomy 8: 161-179

Jones J, Hoogenboom G, Porter C, Boote K, Batchelor W, Hunt L, Wilkens P, Singh U, Gijsman A, Ritchie J 2003. The DSSAT cropping system model. European Journal of Agronomy 18: 235-265

Kassie BT, Asseng A, Porter CH, Royce F 2016. Performance of DSSAT-Nwheat across a wide range of current and future growing conditions. Field Crops Research 81: 27-36

Keating BA, Carberry PS, Hammer GL *et al.* 2003. An overview of APSIM, a model designed for farming systems simulation. European Journal of Agronomy 18: 267-288

Kersebaum KC 2007. Modelling nitrogen dynamics in soil-crop systems with HERMES. Nutrient Cycling in Agroecosystems 77: 39-52

Kersebaum KC 2011. Special features of the HERMES model and additional procedures for parameterization, calibration, validation, and applications. Ahuja, L.R. and Ma, L. (eds.). Methods of introducing system models into agricultural research. Advances in Agricultural Systems Modeling Series 2, Madison (ASA-CSSA-SSSA): 65-94

Li C, Cao W, Zhang Y 2002. Comprehensive Pattern of Primordium Initiation in Shoot Apex of Wheat. ACTA Botanica Sinica: 273-278

Maiorano A, Martre P, Asseng S, *et al.* 2017. Crop model improvement reduces the uncertainty of the response to temperature of multi-model ensembles. Field Crops Res 202: 5-20

Martre P, Jamieson PD, Semenov MA, Zyskowski RF, Porter JR, Triboi E 2006. Modelling protein content and composition in relation to crop nitrogen dynamics for wheat. Eur. J. Agron. 25: 138-154

Nendel C, Berg M, Kersebaum K, Mirschel W, Specka X, Wegehenkel M, Wenkel K, Wieland R 2011. The MONICA model: Testing predictability for crop growth, soil moisture and nitrogen dynamics. Ecological Modelling 222: 1614-1625

Pan J, Zhu Y, Cao W 2007. Modeling plant carbon flow and grain starch accumulation in wheat. Field Crops Research 101: 276-284

Pan J, Zhu Y, Jiang D, Dai TB, Li YX, Cao WX 2006. Modeling plant nitrogen uptake and grain nitrogen accumulation in wheat. Field Crops Research 97: 322-336

Pirttioja, N.K., Pirttioja, N., Carter, *et al.* 2015. A crop model ensemble analysis of temperature and precipitation effects on wheat yield across a European transect: a crop model ensemble analysis using using impact response surfaces Climate Research 65, 87-105. DOI: 10.3354/cr01322

Priesack E, Gayler S, Hartmann H 2006. The impact of crop growth sub-model choice on simulated water and nitrogen balances. Nutrient Cycling in Agroecosystems 75: 1-13

Ritchie JT, Godwin DC, Otter-Nacke S 1985. CERES-wheat: A user-oriented wheat yield model. Preliminary documentation,

Ritchie S, Nguyen H, Holaday A 1987. Genetic diversity in photosynthesis and water-use efficiency of wheat and wheat relatives. Journal of Cellular Biochemistry: 43-43

Senapati N, Stratonovitch P, Paul MJ, Semenov MA 2019. Drought tolerance during reproductive development is important for increasing wheat yield potential under climate change in Europe. J Exp. Botany, 70 (9): 2549-2560

Senthilkumar S, Basso B, Kravchenko AN, Robertson GP 2009. Contemporary evidence of soil carbon loss in the US corn belt. Soil Sci Soc Am J 73: 2078-2086

Shibu M, Leffelaar P, van Keulen H, Aggarwal P 2010. LINTUL3, a simulation model for nitrogen-limited situations: Application to rice. European Journal of Agronomy 32: 255-271

Soltani A, Maddah V, Sinclair T 2013. SSM-Wheat: a simulation model for wheat development, growth and yield. International Journal of Plant Production 7: 711-740

Spitters CJT, Schapendonk AHCM 1990. Evaluation of breeding strategies for drought tolerance in potato by means of crop growth simulation. Plant and Soil 123: 193-203

Steduto P, Hsiao T, Raes D, Fereres E 2009. AquaCrop-The FAO Crop Model to Simulate Yield Response to Water: I. Concepts and Underlying Principles. Agronomy Journal 101: 426-437

Stenger R, Priesack E, Barkle G, Sperr C 1999. Expert-N A tool for simulating nitrogen and carbon dynamics in the soil-plant-atmoshpere system. In. Land Treatment collective proceedings Technical Session, New Zealand

Stockle C, Donatelli M, Nelson R 2003. CropSyst, a cropping systems simulation model. European Journal of Agronomy 18: 289-307

Stratonovitch P, Semenov MA 2015. Heat tolerance around flowering in wheat identified as a key trait for increased yield potential in Europe under climate change J. Exp Botany 66 (12): 3599-3609

Tao F, Yokozawa M, Zhang Z 2009. Modelling the impacts of weather and climate variability on crop productivity over a large area: A new process-based model development, optimization, and uncertainties analysis. Agricultural and Forest Meteorology 149: 831-850

Tao F, Zhang Z 2010. Adaptation of maize production to climate change in North China Plain: Quantify the relative contributions of adaptation options. European Journal of Agronomy 33: 103-116

Tao F, Zhang Z 2013. Climate change, wheat productivity and water use in the North China Plain: A new super-ensemble-based probabilistic projection. Agricultural and Forest Meteorology 170: 146-165

Tao F, Zhang Z, Liu J, Yokozawa M 2009. Modelling the impacts of weather and climate variability on crop productivity over a large area: A new super-ensemble-based probabilistic projection. Agricultural and Forest Meteorology 149: 1266-1278

Wang E, Engel T 2000. SPASS: a generic process-oriented crop model with versatile windows interfaces. Environmental Modelling & Software 15: 179-188

Webber H, Gaiser T, Oomen R, Teixeira E, Zhao G, Wallach D, Zimmermann A, Ewert F 2016. Uncertainty in future irrigation water demand and risk of crop failure for maize in Europe. Environmental Research Letters 11: 074007

De Wit A, Boogaard H, Fumagalli D, Janssen S, Knapen R, van Kraalingen D, Supit I, van der Wijngaart R, van Diepen CA 2019. 25 Years of the WOFOST Cropping Systems Model. Agricultural Systems 168:154–67. https://doi.org/10.1016/j.agsy.2018.06.018

Yan M, Cao W, C. Li ZW 2001. Validation and evaluation of a mechanistic model of phasic and phenological development in wheat. Chinese Agricultural Science 1: 77-82

Yin X, van Laar HH 2005. Crop systems dynamics: an ecophysiological simulation model of genotype-by-environment interactions. Wageningen Academic Publishers, Wageningen, The Netherlands

Supplementary Protocol S1. Crop model performance and evaluation metrics

The mean squared error (MSE) is the sum of the squared residuals, divided by the number of residuals

$$\mathrm{MSE} = \frac{\sum_{i=1}^{n}(S_i - O_i)^2}{n}$$

MSE can be split into three components:

- The squared bias (SB), which is the squared difference between the mean of the simulated values and the mean of the observed values

$$SB = (\bar{S} - \bar{O})^2$$

- The non-unity slope (NU)

$$NU = (1 - b)^2 \times \left(\sum \frac{(S_i - \bar{S})^2}{n}\right)$$

  Where b is the slope of the least-squares linear regression of simulated on observed values.

- The lack of correlation (LC)

$$LC = (1 - r^2) \times \left(\sum \frac{(O_i - \bar{O})^2}{n}\right)$$

Where r^2 is the square of the correlation coefficient of the least-squares linear regression of simulated on observed values.

Modelling accuracy can be quantified with the Nash–Sutcliffe model efficiency (EF), a skill measure calculated as one minus the ratio of the error variance of the simulated values divided by the variance of the observed values.

$$\mathrm{EF} = 1 - \frac{\sum_{i=1}^{n}(S_i - O_i)^2}{\sum_{i=1}^{n}(O_i - \bar{O})^2}$$

EF is useful for making statements about the skill of a model: for a model that simulates perfectly, EF = 1, while for a model that has the same squared error of simulation as the mean of the measurements, EF = 0. EF is negative for a model that has a bigger squared error than the mean of the measurements. The ability of EF to characterize model performance is also dependent on the variability of the observed values compared to model error.
